# Potentially Harmful Excipients: State of the Art for Oral Liquid Forms Used in Neonatology and Pediatrics Units

**DOI:** 10.3390/pharmaceutics16010119

**Published:** 2024-01-17

**Authors:** Marianne Bobillot, Violaine Delannoy, Alexandre Trouillard, Jean Marie Kinowski, Noelia Maria Sanchez-Ballester, Ian Soulairol

**Affiliations:** 1 Department of Pharmacy, Nîmes University Hospital, 30900 Nîmes, France; marianne.bobillot@aphp.fr (M.B.); violaine.delannoy@chu-nimes.fr (V.D.); alexandre.trouillard@chu-nimes.fr (A.T.); jean.marie.kinowski@chu-nimes.fr (J.M.K.); noelia.sanchez-ballester@umontpellier.fr (N.M.S.-B.); 2 ICGM, Montpellier University, CNRS, ENSCM, 34090 Montpellier, France

**Keywords:** pediatric, neonatology, age-dependent, pharmaceutical excipient, toxicity

## Abstract

The pediatric population exhibits an important age-dependent heterogeneity in pharmacokinetics and pharmacodynamics parameters, resulting in differences in drug efficacy and toxicity compared to the adult population, particularly for neonates. Toxicity and efficacy divergences have been studied for active molecules, but the impact on the pharmacological parameters of excipients remains less well known. To fill this lack of knowledge, several initiatives have been started to gather information on the specific toxicity of excipients, such as the KIDS list or the STEP database. In order to contribute to this much-needed action, in this work, a compilation of the 219 formulations of oral liquid forms prescribed in pediatrics and neonatology units was established based on the summary of product characteristics. Then, for excipients found in more than 10% of the analyzed formulations, a review of their toxicity data was carried out using the STEP database. Finally, for a selection of 10 frequently used liquid forms, the amounts of excipients administered daily were calculated based on the recommended posology in the Summary of Product Characteristics (SPC) and compared with the recommended daily limits proposed by the European Medicine Agency. Pediatrics-adapted formulations are still rare, and it is not always possible to find safe alternatives to drugs containing excipients of interest.

## 1. Introduction

The pediatric population, which includes children from birth to 18 years of age, is very heterogeneous and experiences during its development a large variability of pharmacokinetic and pharmacodynamics parameters. This results in differences in drug efficacy and toxicity not only compared to adults but also within the pediatric population [1,2,3]. This pharmacokinetic variability occurs at every step of the drug path, i.e., absorption, distribution in the organism, metabolism and elimination, for example due to hepatic and renal function immaturity at birth [2,4].

The pharmacodynamics parameters of drugs are also age-impacted; for example, specific drug receptors are expressed differently in newborns and children than in adults, and this expression varies during a child’s development. This results in differences in the concentration of drugs in the blood but also in their specific active site, which influences their pharmacodynamic effect with potentially lower efficacy or increased toxicity. This variability is particularly observed in neonates and especially in preterm newborns, making it necessary to adapt the posology of active substances in this age group. It is important to note that while much information has been gathered on the impact of age variability on pharmacological parameters and the toxicity of active molecules, the safety profile of excipients remains less well known [3,5].

Excipients are components of drug formulations used for a wide range of applications, inter alia to improve the solubility, palatability or physicochemical stability of the active substance, but also to preserve it from microbiological contamination. Excipients are expected to be inactive; however, it has been observed that several of them are associated with toxicity, leading to the definition of excipient of interest (EOI) [6,7,8,9,10].

Excipients have been the subject of particular attention for many years, notably with the creation in 1991 of the International Pharmaceutical Excipients Council (IPEC), which in 1997 drew up guidelines for the pharmaceutical industry concerning the safety evaluation of excipients [6]. Nevertheless, data on the toxicity of excipients in the pediatric population are still scarce and often extrapolated from the adult population.

Several recent initiatives have been taken to study excipients and their possible toxicity in the pediatric population, including neonates, more specifically. The European Medicine Agency (EMA) has published a number of discussion papers and established guidelines for the labelling and package leaflet of medicinal product for EOI, concerning both adult and pediatric populations [7]. In addition, since 2001, a major European study on the toxicity of excipients in neonatology has been underway: the European Study of Neonatal Exposure to Excipients (ESNEE). A priority list of excipients considered toxic has been established, and further studies are in progress to measure the exposure of neonates to these excipients [8,9].

Also, based on specialized literature, censuses are progressively set up to identify the molecules of concern, such as the “KIDS list” (Key Potentially Inappropriate Drugs) published in 2020 (containing active substances as well as excipients) [10]. Another initiative specifically targeting excipients is the STEP Database (Safety and Toxicity of Excipients for Pediatrics), which is a database developed since 2012 gathering several studies analyzing the toxicity of excipients in children and newborns as well as in the adult population [11,12,13].

These findings have allowed the development of legislation concerning the formulation and use of pediatric and neonatology medication, with obligations to indicate the presence and quantity of EOI in drugs leaflets, and contraindications for certain excipients that have shown to be toxic in children. For instance, in 2011, the labeling of KALETRA oral solution (Lopinavir/Ritonavir) was changed because the amount of ethanol exceeded the tolerance threshold in neonatology, whereas it was previously authorized for neonates and children [14].

Yet, there is still a lack of drugs developed specifically for children, and then pediatric patients are often treated with off-label medications [15,16,17,18].

Regarding drug administration, the oral route is generally preferred when possible, as it is non-invasive. In particular, in pediatric and neonatal units, oral liquid forms are widely used, as solid forms are contraindicated before the age of 6 due to anatomical and neurological immaturity [13]. In addition, oral liquid forms (i.e., syrups, suspensions and ready-to-use solutions or reconstitutable powder or granules) offer advantages for the pediatric population, as they enable the dose administered to be adjusted (with weight-adapted syringes or spoons) during the child’s growth. However, oral liquid forms present an increased risk of misuse with errors in sampling the volume to administer or a risk of confusion between the prescribed dose and the volume [19,20,21]. Furthermore, their formulation can be complex, requiring the addition of various excipients to enable processing, to improve their physicochemical stability or to prevent a microbiological contamination. In particular, ready-to-use forms frequently contain preservatives, some of which are known to induce toxicity.

This study therefore involved investigating the qualitative and quantitative composition of a wide panel of pediatric liquid oral medications in order to identify the most common excipients found in these formulations. Then, considering the toxicity data and recommended daily limits available for these excipients, a screening was carried out to assess whether the excipient compositions of the pediatric liquid oral forms investigated were suitable for this population.

## 2. Materials and Methods

A compilation of ready-to-use (or to be reconstituted) oral liquid forms, in single dose (sachets) or multi-dose (vials), used in French pediatric and neonatal hospital units was established. Their qualitative and quantitative excipient composition was recorded using the summary of product characteristics (SCP) of each drug product, and when data were incomplete, the pharmaceutical laboratory was contacted (Appendix A). The compositions of the generic formulations, when available, were also recorded. This list made it possible to identify the most common excipients in these formulations, i.e., those representing more than 10% of the total. For these excipients, a review of the toxicity data and daily recommended limits for the pediatric population was completed, respectively, by using the STEP toxicity database and guidelines from the Committee for Human Medicinal Product of EMA.

Finally, a retrospective two-year survey of oral liquid forms commonly used between 2020 and 2021 in our pediatric departments, i.e., general pediatrics, pediatric psychiatry, neonatology and neonatal intensive care units, was realized. Ten of the most widely prescribed drugs were selected. The administered amounts of the EOI were calculated by following the posology recommended by the SCP, and in the UpToDate database, when necessary. Then, the cumulative administered doses were compared with the daily recommended limits to identify possible overdoses of excipients at normal dose ranges of administration.

## 3. Results

### 3.1. Cartography

A total of 219 medicines concerning 123 active substances were analyzed, of which 137 (63%) were ready-to-use and 82 (37%) were drugs to be reconstituted (powder or granules for oral solutions or suspensions). The total number of excipients was 140 and the medicines contained on average seven excipients (see Appendix A).

Sixteen of these excipients were present in more than 10% of the formulations. The proportion and function of these excipients in the formulations are shown in Table 1.

The analysis of the main excipients according to the type of formulation (ready-to-use or to be reconstituted) shows that the excipient repartition can be influenced by this parameter. Thus, while solvents, preservatives, sweeteners, and buffering agents are the most represented excipients in the ready-to-use forms, viscosifying and suspending agents, as well as sweeteners, predominate in the formulations to be reconstituted. More precisely, in terms of the proportion of preservatives, 45% of the ready-to-use forms contained methyl parahydroxybenzoate and 35% contained propyl parahydroxybenzate (compared to less than 10% in the to be reconstituted forms). Similarly, 15% of the ready-to-use forms (21 of 137 formulations) contained sodium benzoate compared to 44% (36 of 82 formulations) of the forms to be reconstituted.

### 3.2. Toxicity Review of the Major Excipients

Among the 16 major excipients, some have been associated with specific toxicities in both thes pediatric and adult populations. The main toxicities and proposed daily intakes are reviewed below.

#### 3.2.1. Solvents

Although water is the solvent of preference, the solubility of most of the active ingredients is poor in water, making it necessary to use other solubilizing agents and co-solvents [22]. Water used for oral liquid medication must comply with the European Pharmacopoeia, and its manufacturing process should guarantee its microbiological quality according to the purified water monograph (04/2018:0008).

##### Propylene Glycol

Propylene glycol is a viscous, clear, colorless, hygroscopic liquid that is miscible with water and ethanol (01/2017:0430) [23]. It is a stabilizing agent for substances hardly soluble in water but also has antimicrobial preservatives and humectant properties. Its metabolism depends on the hepatic alcohol dehydrogenase and aldehyde dehydrogenase enzymes. These enzymes do not mature before the age of 4, leading to different adverse effects such as hypotension, arrhythmia, or hemolysis when administered to infants or children due to the accumulation of toxic metabolites [24,25].

These enzymes are also involved in the metabolism of ethanol, which can increase the risk of toxicity when used together by a saturation mechanism particularly for aldehyde dehydrogenase [26,27,28].

In the STEP database, several cases of toxicity in pediatric patients have been summarized, such as hepatic toxicity, arrhythmia, seizures, abnormal electroencephalogram abnormalities, central nervous system depression and severe metabolic acidosis [29,30].

In the literature, the main toxicity observed is neurological, but hyperosmolarity, lactic acidosis, raised plasma creatinine and bilirubin are also present [31,32]. According to the EMA, the maximum recommended dose of propylene glycol is 1 mg.kg^−1^ per day for neonates up to 28 days or 44 weeks post-menstrual age for preterm neonates; 50 mg.kg^−1^ per day between 1 month and 4 years; and 500 mg.kg^−1^ per day beyond 5 years old [33].

##### Ethanol

Ethanol is one of the excipients of concern to international health regulatory agencies. It is widely used as a solvent but also as a co-saver, antimicrobial preservative and extraction solvent in herbal medicines. It is rapidly absorbed, mainly in the duodenum and jejunum, and partly in the stomach. It is a small molecule with a molecular weight of 46 g.mol^−1^; therefore, it passes the blood–brain barrier well, which explains why it has major neurological toxicity [34,35].

Ethanol is primarily metabolized in the liver into acetaldehyde, which is oxidized to acetate. The main cause of toxicity is the accumulation of acetaldehyde [36].

The STEP database contains several reports of neurological toxicity, brain damage, loss of consciousness, hypoglycemia, hypoglycemic coma, acidosis and hydro-electrolytic alterations. A very high intake can lead to stupor, coma, respiratory depression and cardiovascular collapse. Hypoglycemic seizures may also occur in children [35]. In 2018, the EMA published guidelines regarding the package leaflet for ethanol-containing medicines, which made no distinction between the adult and pediatric populations with regard to the dose limit intake. Even though the acceptable daily intake (ADI) has been set for acute central nervous system toxicity after a single dose administration, the effect of long-term exposure to ethanol in children has not been yet evaluated [37,38].

In this study, the previous EMA recommendations from 2014 were used to assess ethanol use in pediatric patients. It was proposed to avoid its administration before the age of 2 years and to establish a maximum dose of 6 mg.kg^−1^ between the ages of 2 and 5 years and 75 mg.kg^−1^ above 6 years old [37,38]. These limits were calculated on the basis of an increase in blood alcohol concentration (BAC) of 0.01 g.L^−1^ for children under 5 years old and 0.125 g.L^−1^ for children over 5 years old, which must not be exceeded [39].

##### Glycerol

Finally, glycerol is used as a solvent, sweetener, viscosity enhancer and antimicrobial preservative in oral solutions and suspensions. It is one of the most employed solvents due to its good suspending activity and preferable taste [16]. Generally, the toxic effects reported in the pediatric population are gastrointestinal and metabolic toxicity, such as mucositis in the stomach, diarrhea, and electrolyte disturbances, particularly when it is used at high concentrations [16]. The STEP database reports only one case of severe hypoglycemia during oral use in children [40].

Gastrointestinal side effects, notably transit acceleration and diarrhea, can be responsible for the lack of absorption of other drugs or food. For example, a study showed that the concomitant use of glycerol taken orally decreased significantly the absorption of several drugs such as omeprazole, cimetidine, and loperamide in children [41].

Neurological effects such as acute encephalopathy have also been reported in the literature when high concentrations were used in adults [42]. This neurological toxicity may be explained by a reversible relaxation of the blood–brain barrier due to the osmotic property of glycerol, which has been observed in animal models [43,44].

Based on these observations, a maximum of 10 g per dose in oral forms has been set, above which the presence of glycerol must be notified on the label and packaging [7]. However, this limit is not specific to pediatric use.

#### 3.2.2. Antimicrobial Preservatives

##### Parabens

From the paraben’s family, methyl parahydroxybenzoate (methyl paraben) and propyl parahydroxybenzoate (propyl paraben) are the most widely used antimicrobial preservatives, as they are effective over a wide range of pH values and present a broad spectrum of antimicrobial activity. Parabens are present in 82% of the oral medications identified. However, no clinical data concerning children were found in the STEP database. In the general population, cases of allergy, anaphylactoid reaction and papulopustular rosacea have been reported after oral ingestion of these substances. In the literature, methyl and propyl parahydroxybenzoate have been shown to bind to estrogen receptors, and propyl paraben has been associated with reduced spermatogenesis and serum testosterone levels as well as endocrine perturbations [45].

They can also produce a cross-hypersensitivity reaction in patients allergic to aspirin due to the structural similarity of their main metabolite (hydroxyparabenzoic acid) and acetylsalicylic acid [34,46].

On those bases, the European Food Safety Authority (EFSA) established an acceptable daily intake (ADI) of no more than 10 mg.kg^−1^ for the sum of methyl paraben, ethyl paraben and propyl paraben. For propyl paraben specifically, an ADI of 2 mg.kg^−1^ (in adult and pediatric populations) was set by the EMA for its specific side effects with no specific limit in pediatrics due to the lack of pharmacokinetic data in children [45,47].

##### Sodium Benzoate

Sodium benzoate is, after parabens, the most used preservative in oral liquid forms. In premature neonates and newborn of less than 4 weeks, sodium benzoate is contraindicated, as it presents a risk of metabolic acidosis and jaundice, since it is responsible for the elimination of bilirubin from its metabolic sites [48,49]. Moreover, neonates cannot conjugate benzoates with the amino acid glycine, leading to an accumulation of benzoic acid and a risk of metabolic acidosis and neurotoxicity such as seizures [50]. Some cases of urticaria and toxicity for the respiratory system (coughing and wheezing) are also described in the STEP database after its oral administration in children [51]. The EMA therefore recommends a maximum dose of 5 mg.kg^−1^ per day, but this limit is not specific to pediatric populations [49].

#### 3.2.3. Sweetening Agents

##### Saccharose

Saccharose (sucrose) is widely used and is the second most represented sweetening agent in the oral forms investigated. It is a natural disaccharide that belongs to the category of natural sweeteners. It is hydrolyzed in the intestine by sucrase into two monosaccharides, glucose and fructose. Consequently, the use of saccharose should be avoided in patients with type I diabetes and used with caution in patients with hereditary fructose intolerance (HFI) [52]. The use of saccharose is also not recommended for patients with poor glucose absorption or sucrase or isomaltase deficiency. It can also cause a decrease in the pH of dental plaque and lead to dental caries as well as metabolic issues such as obesity. Therefore, it is not intended for long-term therapy [53].

The EMA has set an ADI of 5 mg.kg^−1^ per day of saccharose in oral medicines for patients with HFI and a recommended ADI of 5 g for diabetic patients [7,54]. Otherwise, no threshold has been set in the general population nor pediatric population. However, particular caution should be taken if a patient is being treated with other medicines containing sorbitol due to the risk of fructose accumulation from both excipients.

##### Sorbitol

Sorbitol is a monosaccharide that is not absorbed into the digestive tract. For this reason, it is considered safe in pediatric patients, although it is a laxative at high doses due to its osmotic properties and can hence cause gastrointestinal disorders like pain, swelling, flatulence, nausea and vomiting as well as osmotic diarrhea [16,34,54]. As sorbitol is metabolized into fructose, it should be avoided in children with fructose intolerance, in whom it can, in isolated cases, cause liver damage leading to coma and even death [53,54,55].

In infants, sorbitol accumulation can lead to diabetic-like complications such as retinopathy and cataracts [55]. The data found in the STEP database are the same: mainly abdominal pain, afebrile diarrhea, modification in the intestinal flora (diminution of streptococci), and carbohydrate malabsorption [56,57]. Like others substances that change gastrointestinal function, it can also modify the absorption of co-administered drugs and thereby confluence with their pharmacokinetics.

Therefore, the maximum amount of sorbitol proposed is 140 mg.kg^−1^ per day for all age groups (above which gastrointestinal toxicity is likely) and 5 mg.kg^−1^ per day for patients with hereditary fructose intolerance (HFI) [47,54,58].

##### Aspartame

Aspartame is an artificial sweetener. Its sweetening ability is more than 150 to 200 times greater than that of saccharose [59]. It is a disaccharide composed of an aspartic acid and a methyl phenylalanine ester. For this reason, it should be avoided in cases of phenylketonuria metabolipathy (PKU), as phenylalanine is harmful for patients suffering from this disease and to pregnant women carrying a child suffering from PKU. For these patients, daily phenylalanine intake must be extremely controlled to avoid symptoms such as developmental delay, stunted growth, seizures, severe intellectual disability and behavioral problems [60].

The STEP database reports that aspartame intake can produce allergic reactions, hypersensitivity, angioedema and urticaria in children. Other adverse effects described are neurological, such as neurotoxicity other than caused by PKU, headache and panic attacks [34,59]. Some described effects are also hypersensitivity reactions, vascular and granulomatous panniculitis, and cross-reaction with sulfonamides [34]. The Food and Drug Administration (FDA), the EMA and the Committee for Medicinal Products for Human Use (CHMP) have set the maximum recommended dose at 40 mg.kg^−1^ per day. Furthermore, aspartame should be avoided in infants under 12 weeks of age [61].

##### Sodium Saccharine

Sodium saccharine is another important artificial sweetener used in pharmaceutical products and toothpastes. Its sweetening power is around 300 times greater than that of saccharose. Animal studies suggest that it has a carcinogenic effect at high doses, but this has not been confirmed by human clinical data [53,62].

No clinical data on the use of sodium saccharine are reported in the STEP database, but animal data show bladder cancer in rats and memory loss [62,63]. It is generally considered safe for the human population; however, the food industry has set a limit as for any sweetening agent. The European Commission has set the acceptable daily ingestion for general populations in food at 5 mg.kg^−1^ per day. It is important to note that there is no specific limit for pharmaceutical products [64].

#### 3.2.4. Buffering Agents and Antioxidants

Citric acid and sodium citrate are widely used as pH buffers in oral formulation, as they are present in more than 50% of the drugs under study. They are not particularly associated with toxicity in the literature, and the STEP database gives no toxicity results. No ADI has been set for these excipients.

Regarding the sodium hydroxide, no cases of toxicity associated to its use were found in the literature or in the STEP database. This excipient is therefore not considered to be an excipient of interest (EOI), and no ADI has been set.

#### 3.2.5. Viscosity-Increasing Agents

Xanthan gum is a natural excipient used to increase the viscosity of syrups and other forms. No data were found in the pediatric population using the STEP database, but data collected in adults show gastrointestinal effects such as a laxative effect, diarrhea and flatulence [65]. Xanthan gum is considered harmless and is classified as a GRAS (Generally Regarded As Safe) excipient by the FDA.

Colloidal silicon dioxide is widely used in pharmaceutical products as an anticaking or suspending agent, emulsion stabilizer or viscosity enhancer. It is considered to be a non-toxic and non-irritant excipient by the oral route. The STEP database only contains data on silica exposure in adults, which has been associated with auto-immune vasculitis, esophageal, gastric cancer, and kidney diseases. However, these effects are linked to exposure to silica in the environment and not in food or drugs. As a pharmaceutical excipient, colloidal silicon dioxide is considered safe, and no ADI has been set for this excipient [66].

#### 3.2.6. Sodium Salts

Sodium salts can be present in formulations under several forms (benzoate, citrate, etc.). Its labeling as an EOI (excipient of interest) comes from the fact that an important intake of sodium has repercussions on blood pressure and homeostasis. Thus, for a medicine labelled “sodium-free”, a threshold below 1 mmol per dose (23 mg) has been set; otherwise, the amount of sodium must be mentioned in the package leaflet [7].

A summary of the main toxicities and maximum doses for all the excipients analyzed in this section is presented in Table 2.

### 3.3. Exposure to Excipients of Interest

In order to analyze more precisely the quantities of excipients administered with the recommended daily allowances, a selection of 10 of the most prescribed medicines was established. Excipients with no described toxicity or no recommended daily intake were not considered in this analysis.

#### 3.3.1. Acetaminophen

Acetaminophen (Doliprane^®^) 2.4% liquid suspension (Opella Healthcare—Sanofi) is a widely used analgesic authorized for pediatric use from 3 to 26 kg, i.e., from birth to 9 years of age, according to the SPC. No generic brands of this formulation have been developed in France. The recommended posology for newborns and children is 10 to 15 mg.kg^−1^ per 6 to 8 h according to UpToDate [60].

The formulation of Doliprane^®^ contains the following EOIs: sorbitol at 500 mg.mL^1^, sodium benzoate at 3 mg.mL^−1^ and sodium saccharin (amount not specified).

The calculation of the administered amount of each EOI as a function of concentration according to the different possible administration schemes is described in Table 3.

Table 3 shows that the daily amount of sorbitol exceeds the ADI for every posology scheme in all age categories. Furthermore, sodium benzoate levels are too high in the general pediatric population for the 15 mg.kg^−1^ schemes, and this excipient is contraindicated in neonates.

Acetaminophen is also available as a liquid solution in sachets of Doliprane Liquiz^®^ at 200 and 300 mg (Opella Healthcare—Sanofi) indicated for children above 11 kg (18 months). Doliprane Liquiz^®^ contains sorbitol (500 mg.mL^−1^ i.e., 4150 mg per 200 mg sachet and 6250 mg per 300 mg sachet), sodium benzoate (25 mg per 200 mg sachet and 38 mg per 300 mg sachet), glycerol and xanthan gum.

The recommended posology for 200 mg sachets is one sachet every 6 h for children weighing 11–16 kg; one sachet every 4 h for children weighing 17–20 kg and up to two sachets every 4 h for children weighing 21–25 kg. With this posology scheme, the amount of sodium benzoate administered is between 8.8 and 9.5 mg.kg^−1^ per day. The amount of sorbitol administered is between 1038 and 1581 mg.kg^−1^, which exceeds the daily limits.

For the 300 mg sachets, the posology scheme is similar: up to four sachets per day for children between 16 and 24 kg, up to six sachets per day for children weighing 25 to 30 kg and up to eight sachets per day for children between 31 and 48 kg. With this scheme, the quantities of sodium benzoate are below the limit of 5 mg.kg^−1^ for the 16 to 24 kg category (3.2 to 4.9 mg.kg^−1^) but exceed it for other weight categories (up to 9.8 mg.kg^−1^). The amounts of sorbitol are between 1041 and 1613 mg.kg^−1^ for all weight categories.

Acetaminophen also exists in the form of oral solutions as Efferalganmed^®^ oral solution and Dolko^®^ (Therabel). The Dolko^®^ oral solution contains methyl and propyl parahydroxybenzoate (unprecise amounts) and saccharose (0.2 g per 1 kilogram graduation). According to the SPC, this formulation is suitable for patients from 3.5 kg (i.e., at birth) to 12 kg (approximately 24-month age), with a maximum amount of 2.4 g of saccharose for 12 kg patients, which is below the 5 g limitation for diabetic patients. However, this amount exceeds the limit of 5 mg.kg^−1^ for patients with HFI.

In the case of Efferalganmed^®^, the formulation contains 0.66 g of saccharose per 4 kg graduation and 144 mg of propylene glycol per 100 mL (1.44 mg.mL^−1^) in the aroma as well as benzyl alcohol.

This formulation is suitable for children from 4 to 32 kg (above 1 month of age) with a recommended posology of 15 mg.kg^−1^ every 6 h, i.e., a maximum of 2 mL.kg^−1^ per day. The maximum amount of saccharose administered is for the mass interval of 4 to 32 kg containing 0.66 g, every 6 h (i.e., 21.12 g), and the amount of propylene glycol is 2.88 mg.kg^−1^, which is less than the limit of 50 mg.kg^−1^ set for children older than 1 month.

Acetaminophen can be also found in the form of powder for reconstitution as Doliprane^®^ (Opella Healthcare—Sanofi) 100, 150, 200, 300, and 500 mg; as Efferalganmed^®^ 80, 150 and 250 mg (UPSA), and as a generic brand from Arrow (300 mg).

According to the SCP, all these formulations are indicated for children between 5 and 50 kg, i.e., from 2 months to approximately 15 years old. The recommended posology is 60 mg.kg^−1^ per day, divided into four or six intakes, i.e., 15 mg.kg^−1^ every 6 h or 10 mg.kg^−1^ every 4 h with an upper limit of 3 g per day.

Doliprane^®^ sachets contain sodium benzoate and saccharose. Efferalganmed^®^ contains sorbitol, sodium benzoate, sodium saccharin and aspartame (only for doses of 150 and 250 mg). The generic formulation (Arrow) includes saccharose and aspartame.

The daily amount of sodium benzoate is 0.6 to 0.9 mg.kg^−1^ with Doliprane^®^ and 5 to 8 mg.kg^−1^ with Efferalganmed^®^, which exceeds the 5 mg.kg^−1^ daily limit.

For aspartame, the daily intake is 2 to 3 mg.kg^−1^ with Arrow and 2 to 4 mg.kg^−1^ with Efferalganmed^®^, which is well below the limit of 40 mg.kg^−1^ limitation. Finally, for Efferalganmed^®^, the daily intake of sorbitol is 12 to 44 mg.kg^−1^, which would therefore be suitable for the general population but would exceed the limit of 5 mg.kg^−1^ for patients with HFI.

The precise maximum daily dose of EOI as a function of the posology of the acetaminophen sachet formulations is shown in Appendix B.

Regarding saccharose, doses are below the 5 g limit in Doliprane^®^ (100 to 200 mg). But an 8 g maximal posology is achieved for Doliprane^®^ 300 and 500 mg, and 6.7 g is achieved with the Arrow 300 mg sachet.

#### 3.3.2. Betamethasone

Betamethasone drops at 0.05% oral solution possess a market authorization for neonates and children in various immunological and inflammatory indications. Organor is the pharmaceutical company which markets the originator drug under the name Celestene^®^, and Arrow, Biogaran, EG and Zentiva market generic drugs.

According to the SCP, the recommended posology is 0.075 to 0.3 mg.kg^−1^ per day for initial treatment and 0.03 mg.kg^−1^ per day for maintenance therapy. The maximum dose of 0.3 mg.kg^−1^ for initial treatment and for the regimen dose for maintenance therapy were used in the calculation.

With regard to the EOI present, propylene glycol appears in all formulations, while sodium benzoate, saccharose and sorbitol were found only in the generic formulations, and glycerol was found only in Celestene^®^.

The concentration of propylene glycol is 310 mg.mL^−1^ for Celestene^®^ and 350 mg.mL^−1^ in the generic formulations; i.e., for initial treatment, a patient receives up to 186 mg.kg^−1^ per day for Celestene^®^ and 210 mg.kg^−1^ per day for the other formulations, which is above the ADI of 50 mg.kg^−1^ recommended for children under 5 years old. For maintenance therapy, the administered amount of propylene glycol is 19 mg.kg^−1^ (Celestene^®^) to 21 mg.kg^−1^ per day, so the ADI for neonates would not be respected, but it is suitable for babies over 1 month old.

For glycerol (600 mg.mL^−1^), the administered amount is 360 mg.kg^−1^ per day during the initial treatment with Celestene^®^. The 10 g threshold would be achieved for a 28 kg patient.

Sodium benzoate (1 mg.mL^−1^) gives a maximum administered amount of 0.6 mg.kg^−1^ per day in generic products, which is below the ADI but contraindicated in neonates. The sorbitol concentration in generic brands is 420 mg.mL^−1^, resulting in an administered quantity of up to 252 mg.kg^−1^ per day during the initial phase of treatment and 31.5 mg.kg^−1^ per day during the maintenance phase. The threshold of 140 mg.kg^−1^ per day can therefore be reached during initial treatment.

All formulations contain saccharose at a concentration of 0.3 g.mL^−1^, leading to a maximum of 0.18 g.kg^−1^ per day during the initial phase (the limit of 5 g would be reached above 28 kg) and 0.023 g.kg^−1^ during maintenance treatment (5 g achieved over 200 kg).

#### 3.3.3. Amphotericin B

Amphotericin B oral suspension (Fungizone^®^ 100 mg.mL^−1^) has a marketing authorization for the treatment of candidiasis in neonates and children at a posology of 50 mg.kg^−1^ per day, which is divided into two or three doses, giving a daily volume of 0.5 mL.kg^−1^. In this formulation, the glycerol concentration is 15 g.100 mL^−1^, i.e., a total 0.075 g.kg^−1^ per day. In order to exceed the maximum dose of 10 g, the patient must weight 133 kg, so the risk of overdose seems unlikely in the pediatric population. At the recommended posology, the daily dose of propylene glycol administered is less than the ADI for every age group, since it is 0.15 mg.kg^−1^ per day.

Ethanol is also included in the composition at a concentration of 4 mg.mL^−1^, so a patient will receive 2 mg.kg^−1^ per day of ethanol. This is not suitable for infants, but the intake is acceptable above 2 years old. Methyl and propyl paraben are present in the Fungizone^®^ formulation at the concentrations of 0.115 g.100 mL^−1^ and 0.035 g.100 mL^−1^, respectively. The total daily amounts of methyl and propyl paraben are 0.575 mg.kg^−1^ and 0.175 mg.kg^−1^, which are below the ADI limits.

Finally, Fungizone^®^ contains 2 mg.mL^−1^ of sodium benzoate, i.e., 1 mg.kg^−1^ per day, and is therefore not suitable for neonates.

#### 3.3.4. Amoxicillin/Clavulanic Acid

Amoxicillin/clavulanic acid in the form of powder for oral suspension 100 mg/12.5 mg.mL^−1^ Augmentin^®^ (GSK) and generic brands (Almus, Arrow, Biogaran, Cristers, EG, Sandoz, Teva, Viatris, Zentiva) is a broad-spectrum antibiotic authorized for use in neonates and children. The recommended posology is 40 to 80 mg.kg^−1^ per day of amoxicillin (with an upper limitation of 3000 mg daily) split in three doses, depending on the severity of the infection. A patient may therefore receive up to 0.8 mL.kg^−1^ per day with a maximum dose of 80 mg.kg^−1^.

Aspartame is used in the composition of Augmentin^®^ and generic formulations of Almus, Arrow, Sandoz, Biogaran, Viatris and Zentiva. These formulations contain 3 to 3.2 mg.mL^−1^ (depending on the laboratories) of aspartame, corresponding to a daily intake of 2.4 to 2.56 mg.kg^−1^ per day. Furthermore, these formulations do not take into account the recommendation to avoid aspartame under 12 weeks old [56].

Sodium benzoate is present in Augmentin^®^ and in the generic formulation of Zentiva at a concentration of 1.7 mg.mL^−1^, so the administered doses range from 0.68 to 1.36 mg.kg^−1^ per day. These doses do not reach the ADI set by the EMA for children, but these formulations are not suitable for neonates.

#### 3.3.5. Azithromycin

Azithromycin powder for oral suspension Zithromax^®^ 40 mg.mL^−1^ (Pfizer) is used to treat specific bacterial infections in children over 3 years old.

The posology recommended in the SPC is 20 mg.kg^−1^ per day with a maximum dose of 500 mg per day (adult dose). This represents 0.5 mL.kg^−1^ per day (maximum 12.5 mL for 500 mg). This formulation contains 774.2 mg.mL^−1^ of saccharose corresponding to an administered dose of 387.1 mg.kg^−1^ per day with a maximum of 9.6 g per day. This product is not suitable for HFI and diabetic patients (with a limit of 5 g daily).

#### 3.3.6. Furosemide

Furosemide oral solution Lasilix^®^ 10 mg.mL^−1^ (Sanofi Aventis) is a diuretic used to treat hypertension and edemas authorized in pediatrics and neonates. The recommended dose is 1 to 2 mg.kg^−1^ per day, i.e., 0.1 to 0.2 mL.kg^−1^. This formulation contains 10.09% volume of ethanol, i.e., 100.9 mg.mL^−1^. The amount of ethanol administered would therefore be between 10.09 and 20.2 mg.kg^−1^ per day. Hence, this formulation is not suitable for children under 6 years old according to the EMA recommendations. This formulation also contains sorbitol, glycerol, methyl and propylparaben; however, the quantities of these excipients are not known.

#### 3.3.7. Captopril

Captopril in the form of an oral solution, Noyada^®^ 5 mg.5 mL^−1^ and 25 mg.5 mL^−1^ (Ethypharm), is an antihypertensive drug used for neonates and children. It is the only conversion enzyme inhibitor suitable for this population. The recommended posology is 0.3 mg.kg^−1^ for the initial dose, which is then increased by titration according to tolerance. The maximum dose is 5 mg.kg^−1^ per day for children and neonates.

This drug contains 0.5 mg.mL^−1^ of sodium benzoate, corresponding to a maximum of dose of 2.5 mg.kg^−1^ per day. This dose is lower than the recommended 5 mg.kg^−1^ per day, but it should not be administered before 4 weeks of age.

#### 3.3.8. Ergocalciferol

Ergocalciferol solution in drops Sterogyl^®^ 2 MUI.100 mL^−1^ (DB Pharma) is the only oral solution of vitamin D2 (other vitamin D drugs contain cholecalciferol) used to treat or prevent vitamin D deficiency in neonates and children. The recommended posology is 1 to 5 drops per day. This drug contains ethanol at a concentration of 14 mg per drop, giving 14 to 70 mg of ethanol per day. For a 3 kg-child, this corresponds to 23 mg.kg^−1^ per day, 3.5 mg.kg^−1^ for a 5-year-old child (approximately 20 kg) and 7 mg.kg^−1^ for a 10 kg child (average weight for a 2-year-old child). This is below the recommended thresholds for a weight of over 12 kg; otherwise, the quantity is too high, and this formulation should be avoided before the age of 2.

#### 3.3.9. Ibuprofen

Ibuprofen oral suspensions Advilmed^®^ 20 mg.mL^−1^ (GSK), Nurofenpro^®^ 20 mg.mL^−1^ (Reckitt Benckiser Healthcare), Antarene^®^ 20 mg.mL^−1^ (Elerte) and a generic from Viatris are non-steroid anti-inflammatory drugs used to treat infections or pain in children aged over 3 months and up to 30 kg (above this weight, it is recommended to use another formulation). The recommended posology is 20 to 30 mg.kg^−1^ per day, which is divided into three to four intakes. Calculations are based on a dose of 30 mg.kg^−1^ per day. The EOIs included in these formulations are saccharose, sodium benzoate, glycerol, propylene glycol and sorbitol for Advilmed^®^. Nurofenpro^®^ carries sodium saccharin and glycerol; Antarene^®^ contains sorbitol, parabens, and sodium saccharin. The Viatris formulation contains sodium saccharin, sodium benzoate and glycerol.

Advilmed^®^ is the only formulation containing saccharose (0.75 g.1.5 mL^−1^) corresponding to 0.75 g.kg^−1^ per day (22.5 g per day for a 30 kg-child) and propylene glycol (4.7 mg.mL^−1^) with a total amount of 7.05 mg.kg^−1^ per day. These amounts are acceptable for the targeted population i.e., children above 3 months. The glycerol in Nurofenpro^®^ is at a concentration of 126 mg.mL^−1^, which corresponds to 189 mg.kg^−1^ per day, or 5.7 g for a 30 kg child, which is below the 10 g threshold. Advilmed^®^ and Viatris also contain glycerol, but the concentration is not known. Sorbitol is present in Advilmed^®^ (100 mg.mL^−1^) with a daily dose of 150 mg.kg^−1^; therefore, it is above the threshold. For Antarene^®^, the amount of sorbitol is unknown.

Finally, regarding sodium benzoate, Advilmed^®^ contains 2 mg.mL^−1^ corresponding to 3 mg.kg^−1^ per day and 0.5 mg.kg^−1^ per day for the Viatris formulation, which is below the limit for children above 3 months of age for both cases.

The summarization of the results for all drugs and excipients studied are presented in Table 4.

## 4. Discussion

Liquid oral forms are easy to administer to the pediatric population and are the major oral forms authorized before 6 years old. A study conducted on both liquid and solid oral forms by Rouaz et al. showed that excipients not recommended for the pediatric population are most commonly used in oral solutions and suspensions (mainly propylene glycol, benzoic acid, polyethylene glycol, polysorbate 80 and sodium benzoate) [34]. In the present study, a total of 219 drugs were studied, and the analysis of the global formulations has found 140 excipients; thus, the further search of knowledge concerning toxicity and tolerance data represents a real challenge. Indeed, for several excipients, the recommended daily limits are not specific to pediatrics (e.g., parabens, saccharose) because of lacking data in this population. In addition, the complexity of these oral liquid forms is confirmed by the average number of seven excipients present in their formulation. Furthermore, of the 16 most commonly found excipients, 10 are known to induce toxicities according to STEP database data.

Regarding the 10 drugs studied (commercialized in the form of 32 formulations), all have marketing authorization for pediatrics, and the majority are also authorized for neonatology. However, the presented results show that approximately 95% of all formulations examined contain at least one EOI. More precisely, among the 10 drugs analyzed, none fully complied with the recommendations concerning ADI and contraindications according to the posology indicated and to the population targeted in SPC.

Looking specifically at acetaminophen (paracetamol) formulations, the composition of Doliprane^®^ 2.4% changed in January 2023. It previously contained Nitasep^®^ (a mixture of parabens) and sorbitol at high levels, and it has been modified to remove the parabens from the formulation. However, the new formulation contains a higher level of sorbitol (500 mg.mL^−1^ compared to 114.31 mg.mL^−1^ in the previous product). Also, it contains sodium benzoate, which is contraindicated in neonates, at a level that may exceed the daily limit for children over 4 weeks of age if used at a dosage of 15 mg.mL^−1^, which is a frequent posology scheme.

In ready-to-use forms, the most common EOIs are preservatives, particularly parabens (45% contain methylparaben and 37% propylparaben) and solvents (27% contain glycerol, 25% propylene glycol and 22% ethanol). While the ADIs for parabens are respected in most of the formulations studied, this is not the case for ethanol and propylene glycol. For instance, acetaminophen liquid suspension (Doliprane^®^) 2.4% is recommended for newborns and children, although it contains sodium benzoate, which is not indicated for neonates. The sachets of powder for oral suspension are not authorized for use in neonatology and also contain EOIs contraindicated for this category of patient (sodium benzoate or aspartame). There is therefore currently no suitable alternative for the oral administration of acetaminophen in liquid form in neonates.

Nevertheless, it is important to note that based on the analysis carried out in this study, the acetaminophen formulations Doliprane^®^ or Arrow^®^ powder appear to be better suited to the pediatric population than those of UPSA, which contain higher quantities of sodium benzoate.

Similarly, results show that for the excipients studied, generic brands of amoxicillin/clavulanic acid from EG, Cristers, or Teva, and ibuprofen formulation such as Antarene^®^, Nurofen^®^ and Viatris seem more adapted to the pediatric population due to the absence of sodium benzoate and sorbitol.

Also, for vitamin D supplementation, which is a standard care for neonates (including preterm) that can be pursued for several weeks, the existence of safe formulation is thus an important consideration. Sterogyl^®^ 2 MUI.100 mL^−1^ oral solution is the only formulation of ergocalciferol authorized for pediatric use, but it contains ethanol. A safer alternative would be to use cholecalciferol instead of ergocalciferol, for instance ZymaD^®^ formulations, which do not contain excipients considered toxic. These results show that a precise prior analysis of the qualitative and quantitative composition of excipients can enable pharmacists and physicians to choose the most suitable formulation especially according to the patient’s age or biological results.

On the other hand, for certain medicines such as azithromycin, there is no alternative formulation or, if there is one, it does not comply with the recommendations, as is the case for the formulations of betamethasone oral drops. In these cases, the potential risk of toxicity associated with the EOI must be considered, taking into account the duration of treatment, the patient’s clinical condition and possible therapeutic alternatives.

Finally, of the 219 formulations studied, 101 (46%) have access to the neonatology market and 38 (37.6%) contain sodium benzoate, 23 (22.8%) contain aspartame and 14 (13.9%) contain ethanol. For instance, Noyada^®^, Lasilix^®^ and Fungizone^®^ are authorized for neonates even though they contain sodium benzoate and/or ethanol. These three medicines may be used for several weeks depending on the child’s medical condition and therefore represent chronic exposure to excipients with neurological toxicity, while no therapeutic alternative is available in oral form to avoid this exposition.

It should be also noted that this study concerning oral liquid forms presents certain bias and limits. Indeed, the analysis of the composition of the drugs was not exhaustive, since some excipients represented in less than 10% of the 219 formulations examined were excluded. This is the case for polysorbate 80 (present in ibuprofen formulations) and benzyl alcohol, which are well-known EOIs and are investigated in the ESNEE study among other toxicity studies [8,67]. Similarly, the daily intakes of excipients were calculated on the basis of the posology recommended in the SCP for each drug. A prospective study of prescriptions would have been more accurate, considering the cumulative quantity of EOI administered and the patient’s characteristics, particularly for preterm neonates. Moreover, the calculations made in this work assume 100% absorption of excipients, ignoring the influence of routes of administration and absorption ontogeny. Actual exposure could be lower considering that excipients as well as active substances can be affected by metabolism. Finally, this inventory only analyzes formulations available on the French market, thus not considering the differences in excipient formulations between countries, as shown in a pan-European study conducted by Nellis et al. [9].

Thus, further work, including a comparison of other formulations from different European countries containing the same active ingredients, could enrich the debate on the use of these drugs.

Another difficulty encountered carrying out this study was the lack of precise quantitative data available in the SPC about EOI for a large part of the studied formulations. Access to these data should be made easier for pharmacists and physicians to help them choose the most appropriate medication in patients or adapt drugs posology. Indeed, for some of the drugs investigated, the daily limits of the EOI would not be reached if the maximal posology of the drug is not administered. This would make it possible to consider the balance between drugs’ efficacy and excipients’ specific toxicity. In addition, a child may be treated with several medicines containing various EOIs which, when used together, may present similar side effects or enhance their respective toxicities: for example, in the case of a concomitant administration of a bethametasone formulation containing propylene glycol and Lasilix^®^ or Fungizone^®^, which contain ethanol. This aspect of exposure to excipients was investigated as part of the SEEN project, which led to a review of the cumulative doses of EOIs administered to polymedicated children [28,68,69].

As previously mentioned, oral liquid forms are complex formulations that require the use of several excipients such as antimicrobial preservatives and sweeteners to improve taste, which increases the risk of toxicity. As an alternative, ready-to-use products have been developed for pharmaceutical compounding using specific raw materials and excipients for oral suspension, such as Inorpha^®^ (Inresa) Ora-Plus^®^ Ora-Sweet^®^ Ora-Sweet^®^ SF Ora-Blend^®^ (Medisca), SyrSpend SF ^®^, and Syrspend Dry^®^ (Fagron). However, these formulations also contain EOIs; for instance, potassium sorbate (a conservative) is found in all of them except Syrspend^®^. Ora-Plus^®^, Ora Sweet^®^ and Ora Blend^®^ also contain parabens, and glycerol is present in Inorpha^®^, Ora Sweet^®^ and Ora Blend^®^ [70]. With the exception of the sodium benzoate contained in Syrspend SF^®^ (less than 0.1%), the quantity of EOI present in these formulations is not specified in the product documents. This information is necessary, however, and must be analyzed in conjunction with the concentration of the various formulations manufactured to verify their suitability for pediatric use.

A potential solution to limit or to avoid the use of excipients that are mandatory for the industry, such as preservatives, could be production on a local scale. Another alternative is the development of solid forms that do not require the use of preservatives and that can be easily administered to young children via new technologies, such as disintegrative tablets or mini-tablets [34]. With this is mind, 3D-medicine printing is an interesting option because it offers several advantages, including the manufacture of dispersible 3D-printlets with dosages adapted to the patients [71,72].

## 5. Conclusions

This study shows that oral liquid formulations are complex, with a varied range of excipients used, and demonstrates the need for ongoing research and the development of pharmaceutical forms adapted to the pediatric population. Indeed, as discussed in this work, most formulations currently administered to children and neonates contain excipients of interest in dose ranges that can induce toxicity with long-term use. For instance, among the 219 formulations studied, 38 (37.6%) contain sodium benzoate, 23 (22.8%) contain aspartame and 14 (13.9%) contain ethanol.

Although daily limits have been set by medical authorities concerning most of the known excipients of interest, these limits are often exceeded during drug administration, especially if several drugs are co-administered. In addition, pediatric-adapted formulations are still rare, and it is not always possible to find safe alternatives to drugs containing excipients of interest. As a result, data on the specific toxicity of excipients of interest is an important area of research and needs to be better documented.

In the meantime, pharmacists and physicians must have access to accurate information on the quantitative composition of drugs in order to optimize patients’ care by selecting the most appropriate drugs or drug combinations, especially in the case of chronic medications.

## Figures and Tables

**Table 1:** Repartition of major excipients in the formulations investigated.

Excipients	Formulations (%) Containing the Excipient	Functionality
Purified water	110 (50%)	Solvent
Sodium saccharin	80 (36.5%)	Sweetening agent
Citric acid	79 (36%)	Buffering agent/preservative
Methyl parahydroxybenzoate (methylparaben)	68 (31%)	Preservative
Saccharose	60 (27%)	Sweetening agent
Sodium benzoate	57 (26%)	Preservative
Propyl parahydroxybenzoate (propylparaben)	56 (25%)	Preservative
Sodium citrate	50 (23%)	Buffering agent
Xanthan gum	47 (21%)	Viscosity increasing agent/emulsifying/suspending
Colloidal silicon dioxide	43 (20%)	Viscosity increasing agent/emulsifying/suspending
Glycerol	39 (18%)	Solvent/humectant
Propylene Glycol	34 (15.5%)	Solvent/humectant
Sodium Hydroxide	34 (15.5%)	Buffering agent
Sorbitol	31 (14%)	Sweetening agent/viscosing agent
Aspartame	30 (13.5%)	Sweetening agent
Ethanol	29 (13%)	Solvent/preservative

**Table 2:** Main toxicities and recFommended acceptable daily intake (ADI).

Excipients	Main Toxicity	Recommended Maximum Doses	Comments
Methyl parahydroxybenzoate	Anaphylaxis, hormone levels perturbation	10 mg.kg^−1^ per day sum of methyl paraben + propyl paraben + ethyl paraben (*EFSA*)	Limit in all age groups
Sodic saccharine	/	5 mg.kg^−1^ per day (European Commission)	Extrapolated from food industry, no specific dose for pharmaceutical products
Citric acid	/	/	/
Propyl parahydroxybenzoate	Anaphylaxis, hormone levels perturbation	2 mg.kg^−1^ per day (and 10 mg.kg^−1^ per day sum of methyl paraben + propyl paraben + ethyl paraben)* (EMA*)	Limit in all age groups
Sucrose (saccharose)	Dental, obesity	5 mg.kg^−1^ per day patients with HFI, 5 g per day for diabetic patients (*EMA*)	Limit in all age groups, no limit in non-diabetic and non- HFI population
Sodium benzoate	Jaundice, metabolic acidosis	5 mg.kg^−1^ per day (>4 weeks old, contraindicated in newborns) (*EMA*)	Mandatory threshold
Sodium citrate	/	/	/
Xanthan gum	Gastrointestinal	/	/
Glycerol	Gastrointestinal	10 g per dose (EMA)	/
Propylene glycol	Neurological, cardiac, gastrointestinal	Neonates (28 days or 44 weeks post menstrual age for preterms) 1 mg.kg^−1^ per day; 1 month–4 years 50 mg.kg^−1^ per day; ≥5 years 500 mg.kg^−1^ per day (EMA)	Mandatory threshold
Sodium hydroxide	/	/	/
Sorbitol	Gastrointestinal, contraindicated if HFI	140 mg.kg^−1^ per day (10 g per 70 kg), 5 mg.kg^1^ per day for patients with hereditary fructose intolerance (HFI) (EMA)	Limit in all age groups, indicative threshold
Ethanol	Neurological	<2 years to be avoided; 2–5 years: 6 mg.kg^−1^; >6 years: 75 mg.kg^−1^ (EMA)	Based on a blood alcohol concentration rise of respectively 0.01 g.L^−1^ and 0.125 g.L^−1^.
Colloidal silicon	/	/	/
Aspartame	Metabolic, contraindicated if PKU (phenylalanine source)	40 mg.kg^−1^ per day, avoid <12 weeks old (EMA)	/

**Table 3:** Daily intake of EOI contained in Doliprane^®^ 2.4% oral suspension depending on posology schemes.

Posology Schema	10 mg.kg^−1^ per 8 h	15 mg.kg^−1^ per 8 h	10 mg.kg^−1^ per 6 h	15 mg.kg^−1^ per 6 h
Daily administered volume	1.25 mL.kg^−1^ daily	1.88 mL.kg^−1^ daily	1.68 mL.kg^−1^ daily	2.5 mL.kg^−1^ daily
Sodium benzoate	3.75 mg.kg^−1^ ▲	**5.64 mg.kg^−1^ ▲**	5 mg.kg^−1^ ▲	**7.5 mg.kg^−1^ ▲**
Sorbitol	**625 mg.kg^−1^**	**940 mg.kg^−1^**	**840 mg.kg^−1^**	**1250 mg.kg^−1^**

Bold results are those that exceed the daily limit. ▲ labelled result marks contraindication in neonates.

**Table 4:** Daily administered amounts of the EOI found in 10 oral formulations in comparison with the recommendations.

	Excipient	Excipient	Aspartame ▲	Sorbitol	Sodium Benzoate ▲	Propylene Glycol	Glycerol	Ethanol ▲	Methyl Paraben	Propyl Paraben	Saccharose	Sodic Saccharine
Drug												
Threshold			40 mg/kg per day, Avoid <12 weeks	140 mg/kg per day	5 mg/kg per day avoid <4 weeks	Neonates 1 mg/kg <4 years 50 mg/kg ≥5 years 500 mg/kg	10 g per dose	<2 years avoid; 2–5 years old 6 mg.kg^−1^; >6 years old 75 mg.kg^−1^	Mixed with ethyl paraben: 10 mg.kg^−1^ per day	2 mg.kg^−1^ per day	5 g per day	5 mg.kg^−1^ per day (food industry)
Acetaminophen 2.4% (authorized in pediatrics + neonatology)		Doliprane^®^		**143 to 286 mg.kg^−1^**		0.85 to **1.7 mg.kg^−1^**			≤2.85 mg.kg^−1^	≤0.49 mg.kg^−1^		
Acetaminophen oral solution (authorized in pediatrics)		Doliprane Liquiz^®^ 200 and 300 mg sachets		**1038 to 1613 mg.kg^−1^**	3.2 to 9.8 mg.kg^−1^							
		Dolko^®^							X	X	Up to 2.4 g per day	
		Efferalganmed^®^				2.88 mg.kg^−1^					Up to 21.12 g per day	
Acetaminophen sachets authorized in pediatrics (above 2 months)		Doliprane^®^ 100, 150, 200, 300, 500 mg			0.6 to 0.9 mg.kg^−1^						1.08 **to 8 g**	X
		Arrow 300 mg	2 to 3 mg.kg^−1^								3.24 to **6.67 g**	X
		Efferalganmed^®^ 80 mg		32 to 44 mg.kg^−1^	5 **to 8.9 mg.kg^−1^**							X
		Efferalganmed^®^ 150, 250 mg	2 to 4 mg.kg^−1^	12 to 18.5 mg.kg^−1^								X
Betamethasone oral solution in drops (authorized in pediatrics and neonatology)		Celestene^®^				**186 mg.kg^−1^**	45 to **360 mg.kg^−1^**				0.023 to **0.18 g.kg^−1^**	
		Arrow		31.5 mg.kg^−1^ to **252 mg.kg^−1^**	0.6 mg.kg^−1 ^ ▲	26 to **210 mg.kg^−1^**						
		BGR										
		EG										
		Zentiva										
Amphotericin B (authorized in pediatrics and neonatology)		Fungizone^®^ children and neonates			1 mg.kg^−1^ ▲	0.15 mg.kg^−1^	0.075 g.kg^−1^	2 mg.kg^−1^ ▲	0.575 mg.kg^−1^	0.175 mg.kg^−1^		
Amoxicillin/Clavulanic acid (authorized in pediatrics and neonatology)		Augmentin^®^	2.4 to 2.56 mg.kg^−1^ ▲		0.68 to 1.4 mg.kg^−1^ ▲							
		Arrow										
		Almus										
		Biogaran										
		Cristers										X
		EG										X
		Sandoz	2.4 to 2.56 mg.kg^−1 ^ ▲									
		Teva										
		Viatris	2.4 to 2.56 mg.kg^−1 ^ ▲									
		Zentiva	2.4 to 2.56 mg.kg^−1^ ▲		0.68 to 1.4 mg.kg^−1^ ▲							
Azithromycin (authorized in pediatrics above 3 years old)		Zithromax^®^									387.1 mg.kg^−1^; max 9.6 g	
Furosemide (authorized in pediatrics and neonatology)		Lasilix^®^		X			X	**9.9 to 19.8 mg.kg^−1^** ▲	X	X		
Captopril (authorized in pediatrics and neonatology)		Noyada^®^			2.5 mg.kg^−1^ ▲							
Ergocalciferol (authorized in pediatrics and neonatology)		Sterogyl^®^						14 to 70 mg per day, **max 23 mg.kg^−1^** ▲				
Ibuprofen (authorized >3 months)		Advilmed^®^		100 to **150 mg.kg^−1^**	2 to 3 mg.kg^−1^	7 mg.kg^−1^	X				0.75 g.kg^−1^, max 22.5 g	
		Nurofen^®^					189 mg.kg^−1^, max 5.7 g					X
		Antarene^®^		X					X	X		X
		Viatris			0.5 mg.kg^−1^		X					X

Results in bold are those that exceed the daily limit. ▲ labeled result marks contraindication in neonates. X marks that the amount of these excipients in the formulation is not indicated.

## Data Availability

Data is contained within the article.

## Appendix A. General Table of Results—Studied Formulations

**Molecule**	**Formulations**	**Pediatric Marketing Authorization**	**Neonates Marketing Authorization**	**Ready to Use**	**To Be Reconstituted**
Hydroxyzine	Syrup Atarax^®^ (UCB)	Yes >30 months	No	X	
Furosemide	Oral Solution Lasilix^®^ (Sanofi)	Yes	Yes	X	
Acetaminophen	Oral Suspension Doliprane^®^ (Opella Sanofi)	Yes	Yes	X	
Acetaminophen	Powder in Sachets Opella, Arrow, Efferalganmed^®^Upsa,	Yes >2 or 3 months	No		X
Acetaminophen	Oral Solution Dolko^®^, Efferalganmed^®^, Dolipraneliquiz^®^	Yes >1 month to 24 months	No	X	
Ciclosporine	Oral Solution Neoral^®^ (Novartis)	>1 year old	No	X	
Mycophenolate	Powder for Oral Suspension Cellcept^®^ (Roche)	>2 years old	No		X
Sirolimus	Oral Solution Rapamune^®^ (Pfizer)	No (18 years old)	No	X	
Tacrolimus	Granules for Oral Suspension Modigraf^®^ (Astellas)	Yes	Yes		X
Cefaclor	Powder for Oral Suspension Alfatil^®^ (Ethypharm)	Yes	Yes		X
Cefpodoxime	Granules for Oral Suspension Orelox^®^ (Sanofi) and Generic Brands Zentiva, Arrow	Yes	Yes		X
Cefpodoxime	Powder for Oral Suspension (Biogaran, Sandoz, Zentiva, Teva, EG, Mylan, Arrow)	Yes	Yes		X
Cefixime	Powder for Oral Suspension Oroken^®^ for Children and Oroken^®^ For Neonates (Sanofi)	Yes	Yes		X
Ciprofloxacine	Granules for Oral Suspension Ciflox^®^ (Bayer)	Yes >1 year old	No		X
Morphine	Solution in Drops and Unidose Oramorph^®^ (Kyowa Kirin Pharma)	6 months	No	X	
Carbamazepine	Oral Suspension Tegretol^®^ (Novartis)	Yes	Yes	X	
Valproic acid	Oral Solution Depakine^®^ (Sanofi) and Generic Arrow	Yes	Yes	X	
Valproic acid	Syrup Depakine ^®^ (Sanofi), Arrow	Yes	Yes	X	
Ethosuximide	Syrup Zarontin^®^ (Essential Pharma)	>3 years old	No	X	
Pyrantel	Oral Suspension Combantrin^®^ (Teofarma)/Helmintox^®^ (Innotech)	Yes >12 kg	No	X	
Amoxicillin	Powder for Oral Suspension Clamoxyl^®^ (GSK) and Generic Brands Arrow, EG, Mylan, Biogaran, Zydus, Teva, Sandoz, Zentiva	Yes	Yes		X
Amoxicillin/clavulanic acid	Powder for Oral Suspension for Children and Neonates Augmentin^®^ (GSK) and Generic Brands (Arrow, Almus, Biogaran, Cristers, EG, Sandoz, Teva Viatris, Zentiva)	Yes	Yes		X
Phenoxymethyl pénicilline	Oral Suspension Oracilline^®^ (UCB)	Yes	Yes	X	
Azithromycin	Powder for Oral Suspension Zithromax^®^ (Pfizer)	Yes >3 years old	No		X
Erythromycin	Granules for Oral Solution Ery^®^ (Recordati)	Yes >3 months	No		X
Metronidazole	Oral Suspension Flagyl^®^ (Sanofi)	Yes	Yes	X	
Nitrofurantoin	Oral Suspension Furadantin^®^ (Goldshield)	Yes >3 months	No	X	
Sulfamethoxazole/Trimetoprim	Oral Suspension Bactrim^®^ (Eumedica)	Yes >6 weeks	No	X	
Rifampicine	Oral Suspension Rifadine^®^ (Sanofi)	Yes >3 months	No	X	
Clarithromycin	Granules for Oral Suspension Zeclar^®^ (Mylan) and Generic Brands (Arrow)	>6 months	No		X
Josamycin	Granules Oral Suspension Josacine^®^ (Astellas)	Yes	Yes >2 kg		X
Linezolid	Oral Solution Zyvoxid^®^ (Pfizer)	No	No	X	
Itraconazole	Oral Solution Sporanox^®^ (Janssen Cilag)	No	No	X	
Posaconazole	Oral Suspension Noxafil^®^ (MSD)	No	No	X	
Voriconazole	Powder for Oral Solution Vfend^®^ (Pfizer)	>2 years old	No		X
Flubendazole	Oral Suspension Fluvermal^®^ (Johnson & Johnson)	Yes >1 year old	No	X	
Amphotericin B	Oral Suspension Fungizone^®^ (Cheplapharm)	Yes	Yes	X	
Oseltamivir	Powder for Oral Suspension Tamiflu^®^ (Roche)	Yes	Yes		X
Valgancicovir	Oral Solution Rovalcyte^®^ (Roche)	Yes	Yes	X	
Fluconazole	Powder for Oral Suspension Triflucan^®^ (Pfizer) and Generic Brands (Arrow, Biogaran, EG, Mylan, Pfizer, Sandoz, Teva, Zentiva)	Yes	Yes		X
Nystatin	Oral Suspension Mycostatine^®^ (Substipharm)	Yes	Yes	X	
Aciclovir	Oral Suspension Zovirax^®^ (Gsk)	Yes	Yes	X	
Abacavir	Oral Solution Ziagen^®^ (Viiv Healthcare)	Yes >3 months	No	X	
Emtricitabine	Oral Solution Emtriva^®^ (Gilead)	Yes >4 months	No	X	
Lamivudine	Oral Solution Epivir^®^ (Viiv), Zeffix^®^ (Gsk)	Yes >3 months	No	X	
Zidovudine	Oral Solution Retrovir^®^ (Viiv)	Yes	Yes	X	
Nevirapine	Oral Suspension Viramune^®^ (Boehringer Ingelheim)	Yes	Yes	X	
Lopinavir/Ritonavir	Oral Solution Kaletra^®^ (Abbvie)	Yes	Yes >14 years	X	
Digoxine	Oral Solution in Drops (Teofarma)	Yes	Yes	X	
Captopril	Oral Solution Noyada^®^ (Ethypharm)	Yes	Yes	X	
Metoclopramide	Oral Solution Primperan^®^ (Sanofi)	Yes >1 year old	No	X	
Cetirizine	Oral Solution in Drops Zyrtec^®^ (UCB) and Generic Brands (Arrow, Biogara)	Yes >2 years old	No	X	
Tramadol	Oral Solution Contramal^®^ (Grünenthal), Topalgic^®^ (Sanofi)	Yes >3 years old	No	X	
Oxycodon	Oral Solution Oxynorm^®^ (Mundipharma)	No	No	X	
Ibuprofen	Oral Suspension Advilmed^®^ (GSK), Nurofenpro^®^ (Reckitt), Antarene^®^ (Elerte), and Generic Brands (Biogaran, Mylan)	Yes >3 months	No	X	
Ibuprofene	Granules to Reconstitute Spifen^®^, Spedifen^®^ (Zambon)	11 year old	No		X
Betamethasone	Oral Solution in Drops Celestene^®^ (Organon) and Generic Brands (Arrow, Biogaran, EG, Zentiva)	Yes	Yes	X	
Methotrexate	Oral Solution (Rosemont)	Yes >3 years old	No	X	
Dasatinib	Powder for Oral Suspension Sprycel^®^ (Bristol Myers Squibb)	Yes >1 year old	No		X
Propranolol	Oral Solution Hemangiol^®^ (Pierre Fabre)	Yes >5 semaines	No	X	
Acebutolol	Oral Solution Sectral^®^ (Cheplapharm)	Yes	Yes	X	
Sildenafil	Powder for Oral Suspension Revatio^®^ (Pfizer)	Yes >1 year old	No		X
L-thyroxine	Oral Solution in Drops (Serb)	Yes	Yes	X	
Sodium perchlorate	Oral Solution in Drops Irenat^®^ (Bayer)	>6 years old	No	X	
Loperamide	Oral Solution Imodium^®^ (Janssen Cilag)	>2 years old	No	X	
Aprepitant	Powder for Oral Suspension Emend^®^ (MSD)	>6 months	No		X
Domperidone	Oral Solution Motilium^®^ (Janssen Cilag)	>12 years old	No	X	
Sucralfate	Sachet for Oral Suspension Ulcar^®^ (Sanofi), Keal^®^ (EG)	>14 years old	CI preterm		X
Sodium alginate/sodium bicarbonate	Oral Suspension Gaviscon^®^ (Reckitt) and Generic Brand Sandoz	Yes	Yes	X	
Sodium alginate/sodium bicarbonate	Oral Suspension in Sachets Gaviscon^®^, Gavisconell^®^, Gavisconpro^®^ (Reckitt) and Generic Brands (Arrow, Biogaran, Cristers, EG, Mylan, Sandoz, Zentiva)	Yes	No	X	
Magnesium hydroxyde/Aluminium hydroxyde	Oral Suspension in Sachets and Bottle Maalox^®^ (Opella)	Yes	No	X	
Midazolam	Oral Solution Unidose Ozalin^®^ (Nordic Pharma)	>6 months	No	X	
Ergocalciferol	Oral Solution Sterogyl^®^ (Dbpharma)	Yes	Yes	X	
Colecalciferol	Oral Solution in Drops and Blisters Zymad^®^ (Mylan), Adrigyl^®^ (Crinex)	Yes	Yes	X	
ADEC vitamins	Oral Solution Uvesterol^®^ (Crinex)	Yes	Yes	X	
Calcifediol	Oral Solution in Drops Dedrogyl^®^ (Dbpharma), Gerda	Yes	Yes	X	
Alfacalcidiol	Oral Solution in Drops Un Alpha^®^ (Cheplapharm)	Yes	Yes	X	
Polyvitamins	Oral Solution in Drops Hydrosol^®^ (Pharmadeveloppement)	Yes >1 year old	No	X	
Indometacin	Oral Suspension Indocin^®^ (Iroko)	>14 years old	No	X	
Diazoxide	Oral Suspension Proglycem^®^ (Eusa)	Yes	Yes	X	
Citric acid + magnesium oxide + sodium picosulfate	Powder for Oral Solution in Sachets Citrafleet ^®^ (Recordati)	No > 18 years old	No		X
Macrogol	Powder for Oral Solution in Sachets Forlax^®^ (Ipsen), Movicol^®^ (Eg) and Generic Brands (Arrow, Biogaran Mylan, Sandoz, Zentiva)	Yes >6 months (4 g) et >8 years old (10 g)	No		X
Macrogol	Powder for Oral Solution in Sachets Transipeg^®^ (Recordati), Ximepeg^®^ (Alfasigma)	No	No		X
Diosmectite	Powder for Oral Suspension Smecta^®^ (Ipsen) and Generic Viatris	>2 years old	No		X
Lactulose	Oral Solution in Bottle Duphalac^®^ (Mylan) and Generic Brands (Biogaran)	Yes	Yes	X	
Lactulose	Oral Solution in Sachets Duphalac^®^ (Mylan) and Generic Brands (Arrow, Biogaran, Mylan, Sandoz, Zentiva)	Yes >7 years old	No	X	
Papaine	Oral Solution Dbpharma	Teenager	No	X	
Phosphoric acid + disodic phosphate	Oral Solution Phosphoneuros^®^ (Recordati)	Yes	Yes	X	
L-carnitine	Oral Solution Levocarnil^®^ (Alfasigma)	Yes	Yes	X	
Ataluren	Granules Oral Suspension Translarna^®^ (Ptc Therapeutics)	Yes	Yes		X
Risdiplam	Powder For Oral Solution Evrysdi^®^ (Roche)	Yes >2 months	No		X
Arginine	Oral Blister (Pierre Fabre)	Yes	Yes	X	
Cyanocobolamine/vitamin B12	Blister Iv/Oral (Opella)	Yes	Yes	X	
Clonazepam	Oral Solution In Drops Rivotril^®^ (Cheplapharm)	Yes	Yes	X	
Felbamate	Oral Suspension Taloxa^®^ (Organon)	Yes >4 years old	No	X	
Gabapentin	Oral Solution Neurontin^®^ (Pfizer)	>3 years old	No	X	
Levetiracetam	Oral Solution Keppra^®^ (Ucb) and Generic Brands (Accord, Arrow, Eg, Mylan, Sandoz)	Yes	Yes	X	
Oxcarbazepine	Oral Suspension Trileptal^®^ (Novartis)	>6 years old	No	X	
Piracetam	Oral Solution Nootropyl^®^ (Ucb) and Generic Brands (Arrow)	>30 kg (9 years old)	No	X	
Riluzole	Oral Suspension Teglutik^®^ (Effik)	No	No	X	
Amitryptilin	Oral Solution in Drops Laroxyl^®^ (Teofarma)	>6 years old énurésie	No	X	
Paroxetin	Oral Solution Deroxat^®^ (Gsk)	>7 years old	No	X	
Fluoxetin	Oral Solution Arrow, Biogaran	>8 years old	No	X	
Escitalopram	Oral Solution in Drops Seroplex^®^ (Lundbeck)	No	No	X	
Citalopram	Oral Solution Seropram^®^ (Lundbeck)	No	No	X	
Clobazam	Oral Suspension Likozam^®^ (Advicenne)	>2 years old	No	X	
Prazepam	Oral Solution in Drops Lysanxia^®^ (Alfasigma)	Yes	Yes	X	
Diazepam	Oral Solution in Drops Valium^®^ (Csp)	>6 months	No	X	
Amisulpride	Oral Solution Solian^®^ (Sanofi), Ohre	No	No	X	
Haloperidol	Oral Solution Haldol^®^ (Janssen Cilag)	>6 years old	No	X	
Loxapine	Oral Solution Loxapac^®^ (Eisai)	>15 years old	No	X	
Chlorpromazine	Oral Solution In Drops Largactil^®^ (Sanofi)	>3 years old	No	X	
Levomepromazine	Oral Solution Nozinan^®^ (Sanofi)	>3 years old	No	X	
Cyamemazine	Oral Solution in Drops Tercian^®^ (Sanofi)	>3 years old	No	X	
Alimemazine	Oral Solution Theralene^®^ (Xo)	>20 kg	No	X	
Zuclopentixol	Oral Solution In Drops Clopixol^®^ (Lundbeck)	No	No	X	
Tiapride	Oral Solution Tiapridal^®^ (Sanofi)	>17 kg	No	X	
Trimebutine	Granules for Oral Suspension Sachet Debridat^®^ (Pfizer)	>5 years old	No		X
Trimebutine	Granules for Oral Suspension in Bottle Debridat^®^	>2 years old	No		X
13C Urea	Powder for Oral Suspension Helikit^®^ (Mayoly Spindler)	No	No		X
Rivaroxaban	Granules for Oral Suspension Xarelto^®^ (Bayer)	Yes	Yes		X
Tranexamic acid	Oral Solution Exacyl^®^ (Cheplapharm), Spotof^®^ (Ccd)	Yes	Yes	X	
Vitamin K	Oral Solution Cheplapharm	Yes	Yes	X	
Cannabidiol	Oral Solution Epidyolex^®^ (Pharmablue)	Yes >2 years old	No	X	
X marks that the amount of these excipients in the formulation is not indicated.					

## Appendix B. Daily Intake of EOI Contained in the Different Paracetamol Sachets Formulations DOLIPRANE®, EFFERALGANMED® and Generic ARROW

DOLIPRANE^®^

100 mg

Weight range	6–8 kg	9–10 kg	11–16 kg	17–20 kg
Posology	100 mg every 6 h	100 mg every 4 h	200 mg every 6 h	200 mg every 4 h
Sodium benzoate: 1.18 mg	0.6 to 0.79 mg.kg^−1^	0.71 to 0.79 mg.kg^−1^	0.6 to 0.86 mg.kg^−1^	0.71 to 0.83 mg.kg^−1^
Saccharose: 0.27 g	1.08 g	1.62 g	2.16 g	3.24 g

150 mg

Weight range	8–12 kg	13–15 kg	16–24 kg	25–30 kg
Posology	150 mg every 6 h	150 mg every 4 h	300 mg every 6 h	300 mg every 4 h
Sodium benzoate: 1.8 mg per sachet	0.6 to 0.9 mg.kg^−1^	0.72 to 0.83 mg.kg^−1^	0.6 to 0.9 mg.kg^−1^	0.72 to 0.86 mg.kg^−1^
Saccharose: 0.4 g	1.6 g	2.4 g	3.2 g	4.8 g

200 mg

Weight range	11–16 kg	17–20 kg	21–25 kg
Posology	200 mg every 6 h	200 mg every 4 h	400 mg every 6 h
Sodium benzoate: 2.3 7 mg	0.59 to 0.86 mg.kg^−1^	0.71 to 0.83 mg.kg^−1^	0.76 to 0.90 mg.kg^−1^
Saccharose: 0.54 g	2.16 g	3.24 g	4.32 g

300 mg

Weight range	16–24 kg	25–30 kg	31–48 kg
Posology	300 mg every 6 h	300 mg every 4 h	600 mg every 6 h
Sodium benzoate 3.60 mg	0.6 to 0.9 mg.kg^−1^	0.72 to 0.86 mg.kg^−1^	0.6 to 0.92 mg.kg^−1^
Saccharose 810 mg	3.24 g	4.86 g	**6.48 g**

500 mg

Weight range	27–40 kg	41–50 kg
Posology	500 mg every 6 h	500 mg every 4 h
Sodium benzoate: 5.9 mg	0.6 to 0.87 mg.kg^−1^	0.7 to 0.86 mg.kg^−1^
Saccharose: 1.3 4 g	**5.36 g**	**8.04 g**

ARROW FORMULATION

300 mg

Weight range	16–24 kg	25–30 kg	31–48 kg
Posology	300 mg every 6 h	300 mg every 4 h	600 mg every 6 h
Aspartame: 12 mg	2 to 3 mg.kg^−1^	2.4 to 2.88 mg.kg^−1^	2 to 3 mg.kg^−1^
Saccharose: 834 mg	3.34 g	5.004 g	**6.67 g**

EFFERALGANMED ^®^

80 mg

Weight range	5–6 kg	7–8 kg	9–12 kg	13–16 kg
Posology	80 mg every 6 h	80 mg every 4 h	160 mg every 6 h	160 mg every 4 h
Sodium benzoate: 9.7 mg per sachet	6.5 to 7.8 mg.kg^−1^	7.2 to 8.3 mg.kg^−1^	6.5 to 8.6 mg.kg^−1^	7.2 to 8.9 mg.kg^−1^
Sorbitol: 48 mg	32 to 38.4 mg.kg^−1^	36 to 41 mg.kg^−1^	32 to 42.7 mg.kg^−1^	36 to 44 mg.kg^−1^

150 mg

Weight range	8–12 kg	13–15 kg	16–24 kg	25–30 kg
Posology	150 mg every 6 h	150 mg every 4 h	300 mg every 6 h	300 mg every 4 h
Sodium benzoate: 16 mg per sachet	5.3 to 8 mg.kg^−1^	6.4 to 7.4 mg.kg^−1^	5.3 to 8 mg.kg^−1^	6.4 to 7.7 mg.kg^−1^
Sorbitol: 36 mg	12 to 18 mg.kg^−1^	14.4 to 16.6 mg.kg^−1^	12 to 18 mg.kg^−1^	14.4 to 17.3 mg.kg^−1^
Aspartame: 7.8 mg	2.6 to 3.9 mg.kg^−1^	3 to 3.6 mg.kg^−1^	2.6 to 3.9 mg.kg^−1^	3 to 3.8 mg.kg^−1^

250 mg

Weight range	13–20 kg	21–25 kg	26–40 kg	41–50 kg
Posology	250 mg every 6 h	250 mg every 4 h	500 mg every 6 h	500 mg every 4 h
Sodium benzoate: 26 mg	5.2 to 8 mg.kg^−1^	6.3 to 7.4 mg.kg^−1^	5.2 to 8 mg.kg^−1^	6.3 to 7.6 mg.kg^−1^
Aspartame: 13 mg	2.6 to 4 mg.kg^−^	3 to 3.7 mg.kg^−1^	2.6 to 4 mg.kg^−1^	3 to 3.8 mg.kg^−1^
Sorbitol: 60 mg	12 to 18.5 mg.kg^−1^	14.4 to 17 mg.kg^−1^	12 to 18.5 mg.kg^−1^	14.4 to 17.6 mg.kg^−1^

Results in bold are the one that overcome the ADI.
